# GDF15: A Hijacked Metabo-Hormone Orchestrating Cachexia and Immunosuppression in Cancer

**DOI:** 10.3390/biom16071070

**Published:** 2026-07-22

**Authors:** Dong-Yang Qi, Yong-Fei Wang, Wei-Lin Jin

**Affiliations:** 1 The First Clinical Medical College, Lanzhou University, Lanzhou 730000, China; qidy2025@lzu.edu.cn (D.-Y.Q.); wyongfei2024@lzu.edu.cn (Y.-F.W.); 2 Institute of Cancer Neuroscience, Medical Frontier Innovation Research Center, The First Hospital of Lanzhou University, The First Clinical Medical College of Lanzhou University, Lanzhou 730000, China

**Keywords:** brain–body communication, cancer cachexia, GDF15, GFRAL, immunotherapy, metaboception, muscle wasting, neuroendocrinology, tumor microenvironment

## Abstract

Cancer is responsible for systemic burdens, most notably cachexia and immunosuppression, that extend far beyond local tumor growth and collectively dictate poor outcomes. While often studied separately, these debilitating syndromes are deeply interconnected. On the basis of emerging evidence of growth differentiation factor 15 (GDF15)’s dual actions in immunity and metabolism, we propose that the stress-responsive hormone GDF15 is hijacked by tumors and repurposed as a central metaboceptive hub that integrates diverse oncogenic stress signals to launch a coordinated, dual pathological cascade. Systemically, it disrupts brain–body communication via glial cell line-derived neurotrophic factor family receptor alpha-like (GFRAL) activation in the brainstem, driving anorexia, metabolic rewiring, and progressive wasting of skeletal muscle and adipose tissue that define cachexia. GDF15 acts as a potent immunosuppressor within the local tumor microenvironment, impairing T cell cytotoxicity and increasing the abundance of regulatory T cells. Crucially, these effects are not parallel but interlinked, forming a self-reinforcing detrimental cycle that accelerates host deterioration and therapeutic failure. This positions the GDF15-GFRAL axis as a unique dual-benefit therapeutic target with the potential to simultaneously ameliorate cachexia, improve patient function and quality of life, and revitalize anti-tumor immunity. Reframing cancer through the lens of a hijacked metabolic sensing system provides an integrated perspective that transforms this formidable challenge of concurrent host wasting and immune evasion into a druggable opportunity, charting a course for novel host-directed therapies that restore systemic homeostasis.

## 1. Introduction

Cancer has long been viewed as a disease characterized by localized cellular proliferation. However, its most devastating clinical manifestations of cancer cachexia and immunosuppression reveal a pervasive systemic dimension [1]. Cancer cachexia is a multifactorial syndrome characterized by progressive wasting of skeletal muscle and adipose tissue. It severely erodes patient quality of life, functional capacity, treatment tolerance, and, ultimately, survival [2]. At the same time, tumors actively sculpt an immunosuppressive microenvironment to evade immune destruction, underscoring the systemic nature of immune dysregulation in malignancy [3]. Historically, these two pillars of cancer morbidity have been investigated in isolation, leaving a critical gap in the understanding of their frequent co-occurrence and synergistic detriment. This void is particularly critical for cachexia, as its severity is a primary determinant of poor outcomes, yet its connection to failing anti-tumor immunity remains underexplored.

The emerging paradigm of brain–body communication provides a unifying framework [4]. Within this framework, the stress-induced hormone growth differentiation factor 15 (GDF15) has emerged as a critical molecular hub. No longer merely a biomarker of cellular distress, GDF15 is hijacked by tumors to function as a pathological metaboceptive hub. It converges diverse oncogenic stress signals and launches a dual offensive. Systemically, it sabotages brain–body communication via its exclusive brainstem receptor glial cell line-derived neurotrophic factor family receptor alpha-like (GFRAL), driving anorexia, metabolic derangements, and peripheral tissue wasting central to cachexia. Locally, it creates an immunosuppressive niche within the tumor microenvironment (TME). In this review, we define GDF15 as a “metaboceptive hub”—a central integration point that senses diverse peripheral metabolic and inflammatory stress signals and translates them into a unified systemic response. Furthermore, we conceptualize GDF15 as a “hijacked metabo-hormone.” This “hijacking” refers to the tumor’s exploitation of a normal physiological stress-response pathway. Rather than a transient elevation to restore homeostasis, tumors drive the chronic, unremitting overproduction of GDF15, thereby transforming a protective signal into a persistent pathological driver.

Historically, circulating GDF15 was predominantly viewed merely as a passive biomarker, reflecting the magnitude of systemic stress, tumor burden, and poor patient prognosis. However, recent mechanistic breakthroughs—particularly the identification of its exclusive central receptor GFRAL and its direct interference with the LFA-1/ICAM-1 axis within the TME—have fundamentally shifted this paradigm. It is now evident that GDF15 is not merely a bystander or an epiphenomenon, but a potent, active driver of disease progression. While its diagnostic and prognostic utility remains intact, its primary pathological significance lies in its active orchestration of both cachexia and immune evasion, thereby elevating it to a compelling therapeutic target.

While previous excellent reviews have extensively detailed GDF15’s role either in metabolic diseases and cachexia or, separately, as an immune biomarker, the main novelty of this manuscript lies in bridging these distinct fields. We propose a unified model where GDF15 acts simultaneously—yet mechanistically distinctly—as a systemic driver of cachexia and a local architect of immunosuppression, creating a self-reinforcing vicious cycle. It first describes the biology of GDF15 as a stress-signal integrator and its exclusive central signaling via the GFRAL-rearranged during transfection (RET) complex. The dual conflict between local immunosuppression within the TME and systemic metabolic hijacking via the brain–body axis is discussed next. Evidence demonstrating how these pathologies fuel each other, creating a feed-forward loop that drives cancer progression, is then presented. Finally, the review explores the promising therapeutic paradigm of targeting the GDF15-GFRAL axis, a strategy with the unique potential to concurrently ameliorate cachexia and reinvigorate anti-tumor immunity. Framing cancer through the lens of a hijacked metabolic sensing system provides an integrated perspective on the formidable challenge posed by concurrent cachexia and immune evasion in malignancy.

## 2. GDF15: A Molecular Stress Integrator at the Brain–Body Nexus

**Figure 1:**
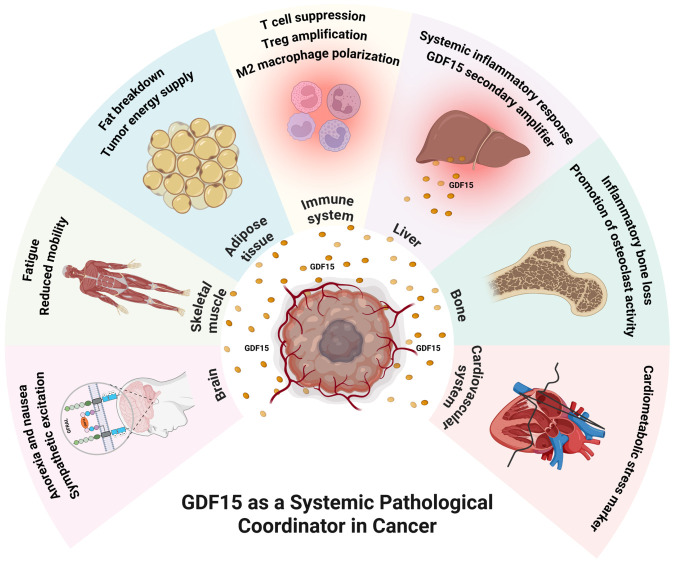
GDF15 acts as a systemic pathological coordinator and induces multi-organ dysfunction in cancer. This schematic delineates how tumor-secreted GDF15 mediates pleiotropic effects across distant organs. GDF15 activates the hindbrain via its exclusive receptor GFRAL, triggering anorexia, nausea, and sympathetic excitation. It promotes fatigue and atrophy in skeletal muscle and induces lipolysis and energy expenditure in adipose tissue. It drives T cell suppression and M2-like macrophage polarization within the immune system, fostering an immunosuppressive microenvironment. The liver serves as a secondary amplifier of circulating GDF15. GDF15 enhances osteoclast activity, promoting inflammatory bone loss. The cardiovascular system expresses GDF15 as a cardiometabolic stress marker. Collectively, these effects illustrate GDF15’s role as a master coordinator of systemic metabolic and immune dysfunction downstream of tumor progression. GDF15, growth differentiation factor 15; GFRAL, glial cell-derived neurotrophic factor family receptor alpha-like. Created in BioRender. Qi, D. (2026) https://BioRender.com/tbqokw3.

GDF15 is a distant member of the transforming growth factor beta (TGF-β) superfamily that functions as a pivotal stress-induced hormone and translates diverse pathological cues into a unified endocrine signal [5] (Figure 1). GDF15 transcription is rapidly upregulated under conditions of cellular stress, including mitochondrial dysfunction, DNA damage, inflammation, and nutrient deprivation, transforming local perturbations into a systemic message [6]. Following its release into the circulation, GDF15 executes its central function by crossing the blood–brain barrier to engage its sole known receptor, GFRAL, which is exclusively expressed in the brainstem’s area postrema and nucleus tractus solitarius [7]. The GDF15-GFRAL-RET axis constitutes the principal conduit through which peripheral metabolic crises are communicated to the central nervous system, establishing GDF15 as a central integrator of brain–body metabolic dialogue.

### 2.1. Physiological Functions: Baseline Roles and Tissue Tolerance

Before exploring its maladaptive roles in malignancy, it is essential to outline the baseline physiological functions of GDF15 to appreciate how it becomes hijacked in cancer. In healthy, non-pregnant adults, GDF15 is expressed at exceedingly low levels in most tissues. Physiologically, it functions primarily as an acute stress-response cytokine rather than a basal homeostatic regulator. Under conditions of acute physiological stress, such as severe viral or bacterial infections, tissue injury, or intense endurance exercise, GDF15 is rapidly secreted to mediate “tissue tolerance”—a defense strategy that protects host tissues from the damaging collateral effects of hyperinflammation without directly affecting pathogen load. Furthermore, GDF15 is highly expressed in the human placenta, where it is vital for maternal–fetal immune tolerance and the physiological regulation of early pregnancy-associated nausea [7]. By transiently modulating appetite and energy expenditure, acute GDF15 signaling preserves metabolic homeostasis during physiological crises. However, in the context of cancer, this normally beneficial, self-limiting protective mechanism is chronically hyperactivated by persistent tumor-derived stress, transforming a physiological safeguard into a pathological driver of systemic wasting and immune evasion.

### 2.2. Transcriptional Integration of Diverse Stress Signals

The induction of GDF15 represents a convergent endpoint for multiple stress signaling pathways originating from the TME [8]. The p53 tumor suppressor pathway is a primary regulator of GDF15 transcription. Pharmacological p53 activation robustly upregulates GDF15, making it a reliable pharmacodynamic biomarker for the p53 pathway activity [6,9]. Beyond genotoxic stress, the integrated stress response (ISR) serves as a master regulator in response to proteotoxic and metabolic insults. Mitochondrial dysfunction activates the ISR kinase protein kinase R to markedly induce GDF15 expression, linking this hormone to mitochondrial stress [10,11]. Nutritional stressors, including amino acid deprivation, also activate the ISR to induce GDF15, triggering conditioned anorexia as an adaptive response to energy imbalance [12]. Inflammatory and senescence-associated stresses represent another major regulation layer. Specifically, systemic inflammation upregulates GDF15 via nuclear factor kappa-light-chain-enhancer of activated B cell (NF-κB) signaling [13], and GDF15 is a core component of the senescence-associated secretory phenotype, where it promotes fibroblast activation and tissue fibrosis [14,15]. Beyond these canonical signaling pathways, GDF15 expression is further fine-tuned by epigenetic mechanisms at the chromatin level, particularly through the dynamic balance between opposing histone modifications. H3K4me3 is a marker of transcriptionally active chromatin that promotes GDF15 transcription. In hepatocellular carcinoma (HCC), the oncogenic transcription factor TCF19 functions as an H3K4me3 “reader”. It selectively recognizes this marker at the GDF15 promoter via its plant homeodomain finger domain, thus enhancing transcriptional activation and driving tumor progression [16]. In contrast, H3K27me3 is a repressive histone marker deposited by the methyltransferase enhancer of zeste homolog 2 that suppresses GDF15 expression by establishing a transcriptionally restrictive chromatin state at its promoter [17]. This antagonistic interplay between H3K4me3 activation and H3K27me3 repression represents a central epigenetic mechanism for calibrating GDF15 expression in response to pathophysiological cues. Together, this multi-input regulatory logic spanning genomic, metabolic, inflammatory, senescence, and epigenetic signals ensures that circulating GDF15 levels faithfully reflect the integrated stress burden across the host.

### 2.3. GDF15-GFRAL-RET Axis: An Exclusive Brainstem Gateway

The biological effects of circulating GDF15 are predominantly mediated through its high-affinity binding to GFRAL, which is selectively expressed in hindbrain regions with a permeable blood–brain barrier [18]. GFRAL itself lacks intrinsic signaling capacity and must act in concert with the co-receptor RET to transduce intracellular signals. Ligand engagement activates RET tyrosine kinase, initiating downstream phosphoinositide 3-kinase/protein kinase B (PI3K/AKT), mitogen-activated protein kinase/extracellular signal-regulated kinase (MAPK/ERK), and cAMP response element-binding protein (CREB) signaling cascades that ultimately reprogram energy homeostasis [19].

Activation of the GDF15-GFRAL-RET axis potently suppresses appetite and induces aversive sensations of nausea, establishing GDF15 as a key mediator of pathological anorexia [20,21]. The anorexic signal can be counterbalanced by other metabolic hormones. For example, glucose-dependent insulinotropic polypeptide antagonizes GDF15/GFRAL signaling by activating inhibitory interneurons, highlighting the nuanced integration of competing signals in the brainstem hub [22,23]. Beyond appetite control, the axis regulates peripheral energy expenditure. GDF15-GFRAL signaling in sympathetic ganglia enhances catecholamine synthesis and promotes lipolysis, increasing thermogenic output [24].

The pathological engagement of the GDF15-GFRAL-RET axis is best exemplified in cancer cachexia, where chronic GDF15 overstimulation of GFRAL drives persistent anorexia and catabolic metabolism [25]. Recent studies have begun mapping the specific neural circuits downstream of GFRAL, revealing that GDF15 and GLP-1 analogs converge on a population of brainstem neurons that project to brain-derived neurotrophic factor-expressing neurons in the medial nucleus of the solitary tract. This pathway is distinct from classical aversive circuits, offering potential avenues for dissociating metabolic benefits from nausea [26].

Importantly, the metabolic effects of some interventions, like metformin, are independent of GFRAL signaling despite elevating GDF15 levels [27]. This indicates that the physiological network is highly complex, and elevated GDF15 does not universally dictate a cachectic phenotype if the central GFRAL axis is bypassed or overridden by other metabolic regulators. Conversely, the therapeutic effects of GDF15 analogs for obesity are wholly GFRAL-dependent [28,29]. This underscores the specificity of the GDF15-GFRAL-RET axis as the dedicated, obligate pathway for the hormone’s central metabolic actions, solidifying its role as a primary driver translating peripheral stress into central behavioral and metabolic responses.

## 3. Local Role of GDF15: Architect of an Immunosuppressive TME

**Figure 2:**
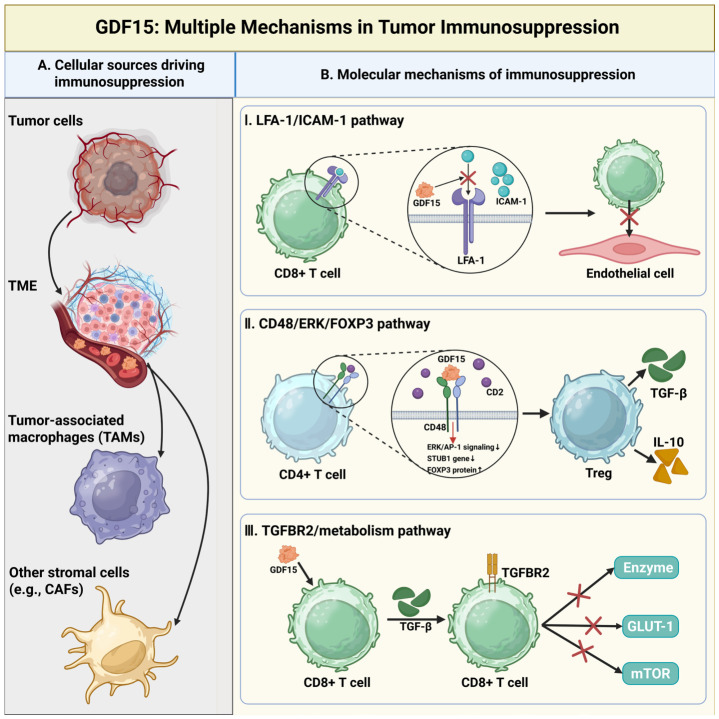
GDF15 orchestrates local immunosuppression in tumor microenvironment. (**A**) GDF15 is secreted into the TME by tumor and stromal cells, including TAMs and CAFs. (**B**) Three core immunosuppressive mechanisms include: (I) impaired CD8^+^ T cell infiltration via LFA-1/ICAM-1 blockade; (II) Treg expansion via the CD48-ERK/FOXP3 pathway with increased TGF-β and IL-10 production; and (III) metabolic suppression of CD8^+^ T cells via the TGFBR2 pathway associated with reduced metabolic enzyme activity, GLUT-1 expression, and mTOR signaling. FOXP3, Forkhead box protein P3; GLUT-1, glucose transporter 1; ICAM-1, intercellular adhesion molecule 1; LFA-1, lymphocyte function-associated antigen 1; mTOR, mechanistic target of rapamycin; TGFBR2, transforming growth factor beta receptor 2; TME, tumor microenvironment. Created in BioRender. Qi, D. (2026) https://BioRender.com/a91ydbl.

Beyond its systemic metabolic functions, GDF15 acts as a potent local disruptor within the TME, actively remodeling it to foster immunosuppression and support malignancy (Figure 2). GDF15 expression is significantly elevated in diverse cancers, correlating with disease progression, immune dysfunction, and poor patient outcomes, and underscoring its value as both a prognostic biomarker and a central mediator of tumor–host interactions [30,31]. This local overexpression is driven by TME-specific stressors, including hypoxia, inflammation, and even physical forces, such as mechanical compression, which in glioblastoma activates the Piezo1 channel to upregulate GDF15 and downstream immune checkpoints [32]. Functionally, GDF15 orchestrates immunosuppression through a multi-pronged strategy. It directly impairs cytotoxic T cell function, expands regulatory immune cell populations, and reprograms stromal cells, collectively establishing a formidable barrier to anti-tumor immunity and promoting resistance against both immunotherapy and chemotherapy [31,33]. Critically, unlike the systemic metabolic effects mediated via the brainstem, these local immunosuppressive mechanisms within the TME operate independently of GFRAL signaling, as GFRAL expression is highly restricted to the hindbrain. Instead, local T cell inhibition and macrophage polarization are mediated through alternative receptors and direct interactions on target immune cells. Within this network, the physical disruption of the LFA-1/ICAM-1 adhesion axis and the polarization of macrophages toward an M2-like phenotype are firmly established mechanisms supported by robust in vivo and clinical data. Conversely, mechanisms such as the metabolic rewiring of T cells and direct dendritic cell impairment represent emerging, context-dependent processes that warrant further validation across diverse tumor microenvironments.

### 3.1. Cellular Sources and Regulation of GDF15 in TME

The cellular origins of GDF15 within the TME are heterogeneous, contributing to the complexity of targeting this pathway. Tumor cells themselves are its primary producers. Tumor-derived GDF15 expression in gliomas and ovarian cancer correlates with malignancy and poor prognosis [34,35]. Single-cell analyses in fibrolamellar carcinoma reveal active GDF15 signaling in tumor epithelial cells, engaging transforming growth factor beta receptor 2 (TGFBR2) on endothelial cells to perturb local communication [36].

Critically, the stromal compartment is a major contributor to GDF15 production within the TME. Cancer-associated fibroblasts (CAFs) in melanoma and head and neck squamous cell carcinoma secrete GDF15. This activates oncogenic PI3K/AKT/STAT3 signaling in tumor cells in a paracrine manner, promoting cancer progression and immunosuppression [37,38]. Similarly, tumor-associated macrophages (TAMs) are induced to produce high GDF15 levels under chemotherapy stress in colorectal cancer (CRC), which then enhances tumor cell chemoresistance [39]. This cooperative secretion from malignant and stromal cells creates a dense, immunosuppressive signaling network that is dynamically regulated by local biochemical and biophysical cues.

### 3.2. Direct Suppression of Adaptive Immunity: T Cell Inhibition via Lymphocyte Function-Associated Antigen 1 (LFA-1) Blockade

The direct inhibition of T cell function is a pivotal mechanism by which GDF15 dismantles anti-tumor immunity. It achieves this, in part, by disrupting the LFA-1-intercellular adhesion molecule 1 (ICAM-1) adhesion axis, which is critical for immune synapse formation and T cell recruitment into tumor tissues [40]. Tumor-derived GDF15 binds to and interferes with this adhesion pathway, effectively blocking T cell infiltration and activation, which is a key mechanism underlying resistance to programmed cell death protein 1 (PD-1)/programmed death-ligand 1 (PD-L1) checkpoint blockade [41].

Clinical evidence supports the therapeutic relevance of this pathway. Neutralizing GDF15 in refractory solid tumors has been shown to restore T cell effector functions, reverse checkpoint inhibitor resistance, and induce sustained anti-tumor responses [42]. Further mechanistic depth comes from gastric cancer studies, where GDF15 from secreted phosphoprotein 1^+^ TAMs engage TGFBR2 on CD8^+^ T cells, driving their exhaustion by upregulating inhibitory receptors [43]. Thus, GDF15-mediated T cell suppression operates through both direct interference with adhesion and indirect promotion of exhaustion, offering multiple nodes for therapeutic intervention.

### 3.3. Amplification of Immunosuppressive Networks: Regulatory T Cell (Treg) Expansion and Macrophage Polarization

In parallel to disabling effector T cells, GDF15 actively expands and activates innate immunosuppressive cell populations. A key such pathway involves the CD48 receptor on T cells. GDF15 binding to CD48 in HCC inhibits the extracellular signal-regulated kinase/activator protein 1 (ERK/AP-1) pathway, leading to E3 ubiquitin ligase STUB1 downregulation. This stabilizes the transcription factor FOXP3 and promotes the differentiation, expansion, and suppressive function of Tregs [44]. A similar Treg-inducing role for GDF15 has been documented in colorectal adenocarcinoma [45].

GDF15 also steers macrophage polarization toward a pro-tumor M2-like phenotype. In prostate cancer, GDF15 activates the PI3K/AKT pathway to induce macrophage M2 polarization, thus promoting resistance to docetaxel [46]. CAF-derived GDF15 in melanoma enhances tumor cell stemness and promotes secretion of CCL18 and TGF-β, inducing macrophage polarization toward an M2 phenotype and accelerating lung metastasis [37]. These effects position GDF15 as a master regulator of the innate immunosuppressive network, coordinating Tregs and M2 macrophages to establish a resilient barrier against immune attack. In addition to these cellular mechanisms, GDF15 directly rewires T cell metabolism to enforce T cell exhaustion, functioning as an immunometabolic checkpoint. Emerging evidence suggests that GDF15 signaling in CD8^+^ T cells carried out via receptors, such as TGFBR2, suppresses glycolytic programs associated with effector function while promoting fatty acid oxidation [43,47]. Although this metabolic shift may support a more quiescent or memory-like state, it comes at the expense of immediate cytotoxic capacity, weakening anti-tumor immunity at a fundamental metabolic level. In this context, GDF15 exerts a “dual strike” on T cells by coupling physical disruption of immune cell adhesion with functional reprogramming of cellular metabolism, ultimately linking nutrient competition, T cell dysfunction, and tumor progression [48,49,50]. Moreover, GDF15 may further reinforce immune evasion by impairing dendritic cell (DC) maturation, antigen presentation, and DC distribution within the TME, thus compromising both the initiation and maintenance of effective anti-tumor immune responses [51]. Consistent with this central immunosuppressive role, therapeutic strategies targeting GDF15, including genetic ablation and antibody neutralization, have been shown to relieve immune suppression and synergize with PD-1 blockade in preclinical models, emphasizing its potential as a critical immune checkpoint [52].

## 4. Systemic Role of GDF15: Hijacking Brain–Body Communication to Drive Cachexia

**Figure 3:**
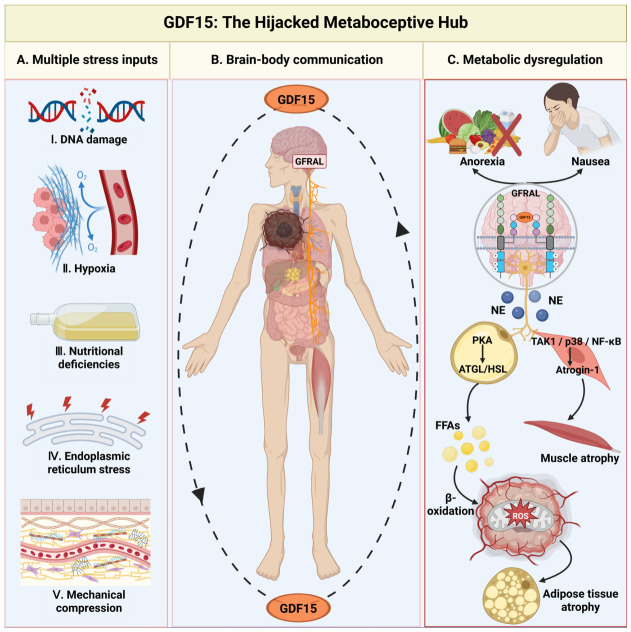
GDF15 drives cancer cachexia via brain–body and peripheral catabolic pathways. (**A**) Multiple stress inputs, including DNA damage, hypoxia, nutritional deficiencies, endoplasmic reticulum stress, and mechanical compression, induce GDF15 overexpression. (**B**) GDF15 activates brainstem GFRAL, suppressing appetite (anorexia/nausea) and promoting sympathetic outflow. (**C**) Peripheral consequences include norepinephrine-driven adipose lipolysis via the PKA-ATGL/HSL pathway, release of free fatty acids that support tumor β-oxidation and ROS generation, and muscle atrophy associated with catabolic signaling. Together, central anorexia and peripheral catabolism drive systemic energy depletion. ATGL, adipose triglyceride lipase; GFRAL, glial cell line-derived neurotrophic factor family receptor alpha-like; HSL, hormone-sensitive lipase; PKA, protein kinase A; ROS, reactive oxygen species. Created in BioRender. Qi, D. (2026) https://BioRender.com/xcniqk0.

The role of GDF15 transforms from a local stress mediator to a potent systemic hijacker when it escapes the confines of the primary tumor and enters the systemic circulation. In cancer and other pathological states, elevated circulating GDF15 levels cause it to act as a hormone-like messenger that pathologically takes over the brain–body axis [53]. The crux of this process is the specific activation of GDF15’s exclusive central receptor GFRAL within key brainstem nuclei in the area postrema and nucleus tractus solitarius [54]. This GDF15-GFRAL dialogue represents a paradigm for understanding systemic syndromes like cachexia. It demonstrates how a peripheral molecular signal can systematically reprogram central regulatory centers, altering whole-body energy metabolism and behavior to favor disease progression (Figure 3). Importantly, preclinical intervention studies differentiate GDF15 from a mere biomarker of systemic inflammation or tumor burden. The observation that neutralizing antibodies against GDF15, or genetic ablation of GFRAL, can actively reverse cachectic weight loss and anorexia confirm the causal role of this axis in driving the cachexia syndrome.

### 4.1. Central Appetite Suppression: From GFRAL Activation to Anorexia

GDF15’s profound effect on the central nervous system’s control of feeding is the most direct manifestation of its systemic hijacking. By binding to GFRAL in the brainstem, GDF15 potently suppresses appetite and induces aversive sensations, leading to the classic symptoms of anorexia and nausea that characterize cachexia [55].

This pathway is a primary driver of pathological anorexia in cancer. Elevated serum GDF15 levels in cachectic patients correlate strongly with appetite loss severity [56]. In preclinical murine models, GDF15 suppresses feeding by activating GFRAL-positive neurons independently of other appetite-regulating hormones like leptin or GLP-1, indicating a non-redundant role [57]. Its effect extends beyond mere caloric reduction to deeper behavioral reprogramming. GDF15 induces a state of visceral malaise and conditioned taste aversion, actively associating eating with negative sensations [21,58].

The axis is also integral to protective emetic reflexes. Hepatic endoplasmic reticulum (ER) stress via the inositol-requiring enzyme 1 alpha/X-box binding protein 1 (IRE1α-XBP1) pathway during chemotherapy upregulates GDF15, which then acts on brainstem GFRAL neurons to trigger nausea. This explains a key component of treatment-related anorexia [59]. The role in establishing aversive memory is further highlighted in food allergy models, where mast cell-induced GDF15 promotes long-term antigen avoidance via GFRAL [60]. Notably, this pathway’s function is not confined to metabolism. The GDF15-GFRAL axis can suppress autoimmune T cell responses in neuroinflammatory contexts, such as experimental autoimmune encephalomyelitis, demonstrating its broader role as an integrator of systemic immune and metabolic signals [61].

Targeting this central nexus thus holds significant therapeutic promise. Preclinical studies consistently show that GDF15-neutralizing antibodies or GFRAL antagonists can effectively reverse anorexia, nausea, and associated weight loss in models of cancer or chemotherapy-induced cachexia [62,63,64].

### 4.2. Peripheral Tissue Wasting Execution: Muscle and Adipose Tissue Catabolism

The systemic impact of GDF15 extends beyond the brain to directly orchestrate the catabolic destruction of peripheral tissues, primarily skeletal muscle and adipose tissue. This process is synergistically linked with the reprogrammed lipid metabolism of the tumor itself, which scavenges lipids to fuel growth, creating a parasitic energy dynamic [65].

While central effects such as anorexia, nausea, and sympathetic excitation are strictly GFRAL-dependent, GDF15 in skeletal muscle drives atrophy through mechanisms that extend past simple nutrient deprivation from anorexia. Certain direct peripheral actions on skeletal muscle and anabolic signaling remain under investigation and may involve indirect pathways or secondary mediators downstream of central GFRAL activation. It can activate a GDF15-GFRAL-sympathetic nerve axis, increasing norepinephrine release in muscle to elevate energy expenditure and directly enhance protein breakdown [66]. Coupled with central anorexia, this sympathetic overdrive creates a powerful catabolic state. GDF15 signaling at the molecular level is implicated in the ubiquitin–proteasome pathway activation, which is a primary engine of muscle protein degradation, potentially through upregulation of muscle-specific E3 ligases, such as atrogin-1 and MuRF1 [67,68]. It may concurrently suppress anabolic signaling, including the IGF-1/PI3K/AKT pathway, leading to reduced protein synthesis and exacerbated muscle wasting [68,69]. In ovarian cancer cachexia models, GDF15 neutralization improved muscle mass and function, with benefits attributable both to improved feeding and to direct feeding-independent effects on muscle, indicating a toxic effect that likely involves these anabolic and catabolic pathways [62].

Stress-induced GDF15 primarily promotes lipolysis and fatty acid oxidation in adipose tissue, depleting energy reserves and disrupting systemic balance [70]. Tumors can obtain large amounts of fatty acids from adipose tissue. These lipids support rapid tumor proliferation and energy supply through reprogramming fatty acid β-oxidation and lipogenic anabolism. The accumulation of lipid peroxidation products can further fuel tumor growth and exacerbate tissue lipotoxicity at the same time [65]. The role of GDF15 in lipid metabolism is context-dependent. While it drives fat loss in cachexia, it may conversely improve hepatic steatosis in non-alcoholic fatty liver disease by mitigating oxidative stress and inflammasome activation [71]. This duality emphasizes GDF15’s function as a homeostatic factor that becomes a driver of wasting when chronically and pathologically activated.

Beyond skeletal muscle and adipose tissue, GDF15 exerts systemic effects on other organ systems, as outlined in Figure 1. In the cardiovascular system, GDF15 is prominently expressed as a cardiometabolic stress marker. Furthermore, in the skeletal system, systemic GDF15 promotes inflammatory bone loss by enhancing osteoclast activity.

This systemic hijacking creates a coherent pathogenic network. Central anorexia reduces energy intake, while peripheral catabolism in muscle and fat accelerates energy expenditure and tissue loss. Interventions that disarm GDF15, such as neutralizing antibodies [72] or receptor decoys [73,74], have shown strong preclinical efficacy in simultaneously blunting both arms of this process, validating the axis as a master regulator of cachectic metabolism.

## 5. The Vicious Cycle: Molecular Feedback Loops Coupling Immunosuppression and Cachexia

**Figure 4:**
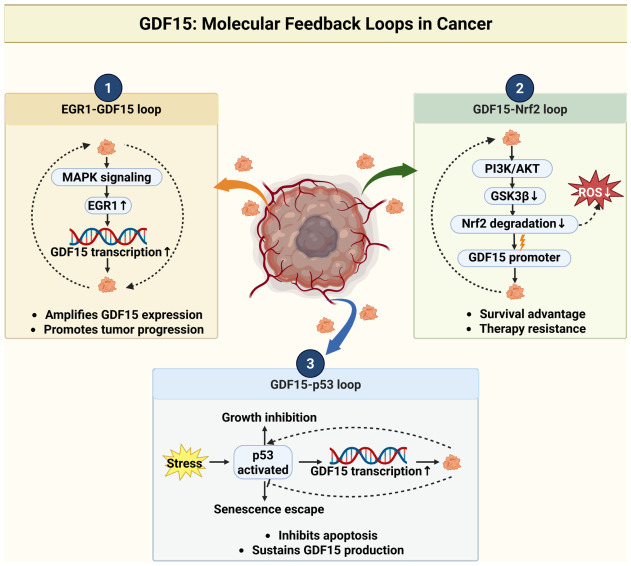
GDF15 participates in molecular feedback loops that support cancer progression. (**1**) In the EGR1-GDF15 loop, GDF15 activates MAPK signaling, increases EGR1 expression, and promotes GDF15 transcription, thereby amplifying GDF15 expression and tumor progression. (**2**) In the GDF15-Nrf2 loop, GDF15 activates PI3K/AKT signaling, suppresses GSK3β, reduces Nrf2 degradation, and enhances GDF15 promoter activity, contributing to reduced ROS levels, survival advantage, and therapy resistance. (**3**) In the GDF15-p53 loop, cellular stress activates p53 and increases GDF15 transcription, forming a regulatory circuit associated with growth inhibition, senescence escape, apoptosis inhibition, and sustained GDF15 production. AKT, protein kinase B; EGR1, early growth response 1; GSK3β, glycogen synthase kinase 3 beta; MAPK, mitogen-activated protein kinase; Nrf2, nuclear factor erythroid 2-related factor 2; PI3K, phosphoinositide 3-kinase; p53, tumor protein p53; ROS, reactive oxygen species. Created in BioRender. Qi, D. (2026) https://BioRender.com/iwo3rzm.

The preceding sections describe the dual, spatially segregated pathologies driven by GDF15. However, the true lethality of this axis in cancer lies in the dynamic interplay between local and systemic effects, which coalesce into a self-reinforcing deleterious cycle. It transforms GDF15 from a mediator of discrete symptoms into the central engine of progressive host deterioration and therapeutic failure (Figure 4). The following framework explores clinical and preclinical evidence to outline the sequential components of this GDF15-driven engine, wherein each step mechanistically fuels the next.

### 5.1. Initiation: Tumor Stress-Driven GDF15 Production

The cycle is ignited by tumor-intrinsic and therapy-induced stresses within the TME, which act as the primary drivers of GDF15 overexpression. This stress response is regulated by transcription factors like early growth response 1 (EGR1), which forms a positive feedback loop with GDF15 to activate the mothers against decapentaplegic homolog 2 and 3 (SMAD2/3), PI3K/AKT, and mitogen-activated protein kinase kinase/extracellular signal-regulated kinase (MEK/ERK) pathways, promoting tumor proliferation, invasion, and epithelial–mesenchymal transition [75]. This phenomenon is widespread. Chemotherapeutics like cisplatin upregulate GDF15 in ovarian cancer [76]. GDF15 mediates sunitinib resistance in melanoma [19] and is highly expressed in drug-tolerant persister cells in breast cancer following eribulin treatment [77]. Furthermore, stromal cells like TAMs are utilized to produce GDF15 under chemotherapy pressure, illustrating a bidirectional stress dialogue within the TME [78]. The initial surge in locally produced GDF15 sets the stage for its dual-pathway offensive.

### 5.2. Local Consequences: Immunosuppression and Therapy Resistance

Secreted GDF15 executes its local immunosuppressive program within the TME. It cripples adaptive immunity by blocking the LFA-1-ICAM-1 axis, preventing T cell infiltration and directly contributing to resistance against PD-1/PD-L1 checkpoint inhibitors [41,79]. It simultaneously fosters an innate immunosuppressive landscape by expanding Tregs via the cluster of differentiation 48/STIP1 homology and U-box containing protein 1/Forkhead box P3 (CD48-STUB1-FOXP3) axis and polarizing macrophages toward an M2 phenotype [44,80,81].

This locally immunosuppressive niche facilitates immune escape and tumor progression and induces broad therapy resistance. GDF15 enhances chemoresistance in CRC by promoting fatty acid β-oxidation [39]. It drives cisplatin resistance in esophageal squamous cell carcinoma via the TGFBR2-AKT-UGT1A pathway [82], and contributes to radioresistance and gemcitabine resistance in breast and ovarian cancers, respectively [83,84]. The resulting unchecked tumor growth and therapeutic failure amplify systemic tumor burden and stress, escalating GDF15 release into the circulation.

### 5.3. Systemic Consequences: Brain-Mediated Cachexia

Once in the bloodstream, circulating GDF15 exploits systemic metabolism via the brain–body axis. It triggers anorexia, nausea, and metabolic rewiring by engaging brainstem GFRAL receptors, leading to the cardinal features of cachexia that include skeletal muscle atrophy and adipose tissue wasting [25,85,86]. This is not a passive consequence of starvation but an active pathological program. GDF15 in HCC drives cachexia while also engaging immunosuppressive pathways [87]. A reciprocal positive feedback loop between GDF15 and the transcription factor nuclear factor erythroid 2-related factor 2 (Nrf2) can maintain this state while promoting oxaliplatin resistance in CRC [88]. Thus, cachexia is established as an active GDF15-driven syndrome that systemically weakens the host.

### 5.4. Immunometabolic Link: Host Debilitation Reinforces Immune Suppression

The cachectic state is not a passive outcome but an active amplifier of the immunosuppressive TME. Host metabolic deterioration directly impairs systemic and local immune competence through several interconnected mechanisms as follows: (1) **Nutrient competition:** Cachexia-induced systemic catabolism depletes glucose, amino acids, and lipids, creating a nutrient-poor milieu that compromises T cell metabolic fitness, proliferation, and effector function [89,90]. (2) **Systemic inflammation:** Chronic wasting is associated with elevated pro-inflammatory cytokine levels (e.g., interleukin 6 (IL-6) and tumor necrosis factor alpha (TNF-α)) that reinforce the expansion and suppressive activity of Tregs and M2-like macrophages [91]. (3) **Metabolite-driven immune reprogramming:** Increased lipolysis and proteolysis release metabolites, such as free fatty acids [92,93] and kynurenine pathway products [94,95], which can directly inhibit T cell function and promote immunosuppressive myeloid cell polarization. (4) **Pharmacokinetic perturbations:** Altered body composition and organ function in cachexia modify drug distribution and clearance, reducing effective concentrations of chemotherapeutic and immunotherapeutic agents within the tumor [96,97].

Thus, the metabolically debilitated host does not merely endure cancer progression but actively perpetuates it by reinforcing local immunosuppression and diminishing therapeutic efficacy.

This self-sustaining engine operates in the following manner: tumor stress (Section 5.1) fuels local immunosuppression (Section 5.2), which permits tumor growth and increases circulating GDF15 levels, which then triggers systemic cachexia (Section 5.3). In turn, the cachectic state creates a metabolically hostile environment that further debilitates immune cells (Section 5.4), closing the loop and accelerating the downhill spiral. Each revolution of this cycle amplifies the next, explaining the inexorable progression of cancer-related morbidity.

### 5.5. Cycle Closure: Positive Feedback Loops Drive Disease Progression

The GDF15-driven vicious cycle is closed and perpetuated through several integrated positive feedback mechanisms. The initial EGR1-GDF15 feedback drives continuous tumor aggressiveness [98]. The GDF15-Nrf2 loop maintains redox homeostasis and chemoresistance [88]. Critically, the progression fueled by local immunosuppression and systemic cachexia leads to increased tumor burden and host stress, which stimulate further GDF15 production from both tumor and stromal cells. This final step ensures the cycle is self-renewing and accelerating.

Furthermore, the GDF15-GFRAL axis functions as a hub for metabolic regulation and a key signaling bridge connecting the nervous, immune, and metabolic systems. Hepatic ER stress induces GDF15 expression during chemotherapy [59]. GDF15 then acts on GFRAL-expressing neurons in the brainstem, leading to nausea and anorexia and potentially activating the hypothalamic–pituitary–adrenal axis, consequently elevating glucocorticoid levels and contributing to a systemic neuroendocrine stress response [99]. The GDF15-GFRAL axis can simultaneously systemically regulate immune cell function through these neuroendocrine pathways, suppressing autoimmune T cell responses in neuroinflammatory settings [61].

In conclusion, the GDF15-driven cycle model redefines cancer progression as a systemic illness orchestrated by a hormone. It links tumor-derived stress to local immunosuppression, cachexia, host debilitation, and back to increased tumor aggression via positive feedback. This framework explains the clinical synergy between cachexia and poor treatment outcomes and positions the GDF15-GFRAL axis as a unique therapeutic lever to simultaneously disrupt both the local and systemic cancer progression engines (Table 1).

## 6. Therapeutic Perspectives: Targeting the Hijacked Hormone to Restore Host Homeostasis

**Figure 5:**
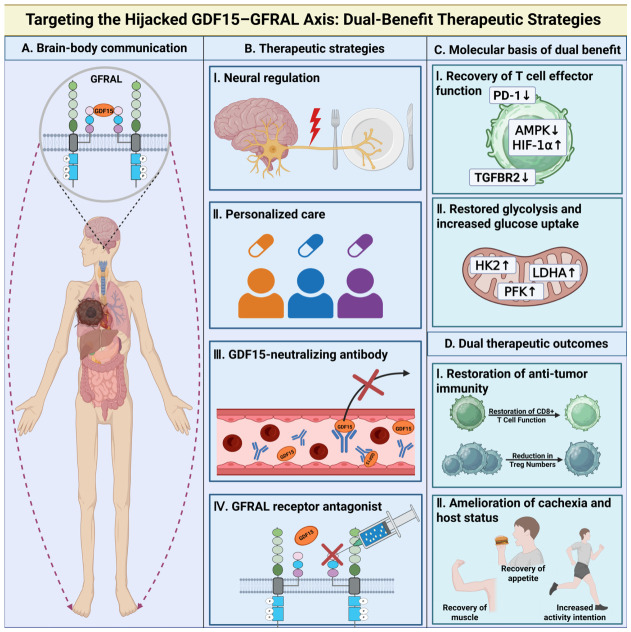
Targeting GDF15-GFRAL offers a dual-benefit therapeutic paradigm. (**A**) Pathological brain–body communication: tumor-derived GDF15 activates brainstem GFRAL, driving anorexia and metabolic dysregulation. (**B**) Therapeutic strategies: neural circuit modulation, biomarker-guided personalized care, GDF15-neutralizing antibodies, and GFRAL receptor antagonists. (**C**) Molecular basis for dual benefit: (I) recovery of T cell effector function characterized by reduced PD-1 and TGFBR2 signaling and increased AMPK/HIF-1α activity and (II) restored glycolysis and glucose uptake with increased expression of HK2, PFK, and LDHA. (**D**) Dual therapeutic outcomes: (I) restoration of anti-tumor immunity, including CD8^+^ T cell function recovery and reduction in Treg numbers and (II) amelioration of cachexia and host status, including recovery of appetite, muscle mass, and physical activity. Targeting this single axis provides synergistic benefits for both cancer immunity and systemic metabolic health. AMPK, AMP-activated protein kinase; GFRAL, glial cell line-derived neurotrophic factor family receptor alpha-like; HIF-1α, hypoxia-inducible factor 1 alpha; HK2, hexokinase 2; LDHA, lactate dehydrogenase A; PFK, phosphofructokinase; Treg, regulatory T cell. Created in BioRender. Qi, D. (2026) https://BioRender.com/t64ev7x.

The consideration of GDF15 as a central hub driving the interconnected pathologies of immunosuppression and cachexia presents a compelling therapeutic rationale. Moving beyond traditional strategies that target tumors or symptoms in isolation [102,103], recalibration of the GDF15-GFRAL axis represents a paradigm shift toward host-directed repair [104] (Figure 5). This approach aims to dismantle the self-reinforcing detrimental cycle by simultaneously addressing its local and systemic arms, offering a unique dual-benefit paradigm with the potential to improve both quality of life and anti-tumor efficacy. For patients suffering from cachexia, this translates to the prospect of prolonged survival and preserved physical function, increased muscle strength, and improved ability to tolerate and benefit from anti-cancer therapies.

### 6.1. Neutralizing Antibodies and Receptor Blockers: From Bench to Bedside

Blocking the ligand–receptor interaction is the most direct strategy to interrupt GDF15 signaling. Preclinical studies robustly validate this approach. GDF15-neutralizing antibodies or genetic silencing reverse cachectic weight loss, muscle atrophy, and fat depletion in various cancer models, often restoring appetite and physical function in patients [62,64,105]. Targeting GDF15 inhibits cachexia development in serine/threonine kinase 11/liver kinase B1 (STK11/LKB1)-mutant non-small cell lung cancer models [104]. Similarly, GFRAL antagonists have shown efficacy in alleviating anorexia and weight loss, confirming the receptor as a druggable node [106,107,108].

Translation to the clinic is underway, with monoclonal antibodies against GDF15 leading the development. Ponsegromab has progressed through phase II human clinical trials, demonstrating promising results in improving weight, appetite, and patient-reported outcomes in cachectic individuals [109,110,111]. Despite these promising preclinical outcomes, transitioning GDF15-GFRAL axis inhibitors to the clinic presents distinct challenges, and potential adverse effects must be carefully weighed. Since GDF15 is critical for mediating tissue tolerance during acute stressors [13], profound and sustained pharmacological blockade of GDF15 could theoretically impair normal stress adaptations, rendering patients more vulnerable to inflammatory injury during acute comorbidities like severe infections or cardiovascular events. Furthermore, recent early-phase clinical trials have highlighted significant pharmacokinetic and pharmacodynamic hurdles. For instance, the first-in-human phase I/IIa trial of the anti-GDF15 monoclonal antibody AZD8853 in patients with advanced solid tumors demonstrated a favorable safety profile with no dose-limiting toxicities. However, the suppression of circulating GDF15 was only transient, and no objective tumor responses or sustained pharmacodynamic effects on peripheral immune cells were observed [112,113]. This underscores that GDF15 elevation may not always strictly correlate with cachexia severity or poor therapeutic response in all clinical contexts, highlighting the necessity for optimized dosing, precise biomarker stratification, and combination strategies. Beyond canonical blockade, upstream suppression of GDF15 transcription via bromodomain and extra-terminal domain family (BET) inhibitors has shown synergistic effects with chemotherapy in preclinical models [114]. These findings underscore the current limitations of GDF15 monotherapy and highlight the urgent need to optimize dosing regimens, enhance antibody binding affinities, and strategically integrate GDF15 blockade with combinatorial approaches, such as ICIs or chemotherapy, to achieve durable clinical efficacy (additional ongoing trials are summarized in Table 2).

### 6.2. Dual-Benefit Paradigm: Simultaneous Cachexia Relief and Immunity Restoration

The most significant promise of targeting the GDF15-GFRAL axis lies in its potential for concurrent dual benefit, directly counteracting both systemic cachexia [115] and local immunosuppression [116]. This strategy moves cachexia therapy from supportive care to an integral part of anti-tumor treatment. By blocking GDF15 signaling, it is possible to reverse anorexia and muscle wasting, thereby improving patient-reported outcomes, physical performance (e.g., grip strength, walk tests), and overall resilience, as well as remodel the immunosuppressive TME. GDF15 blockade is hypothesized to restore anti-tumor immunity through a synergistic, dual mechanism. Directly, it improves immune cell infiltration and function by relieving the local inhibition of the LFA-1/ICAM-1 adhesion axis within the TME. Indirectly, it enhances global immune competence by reversing systemic catabolism, thereby reducing nutrient competition and lowering the systemic inflammatory mediators that would otherwise impair T cell fitness. Preclinical evidence confirms that GDF15 neutralization reinvigorates T cell function, enhances CD8^+^ T cell infiltration, and synergizes with or even reverses resistance to immune checkpoint inhibitors (ICIs) like those used in anti-PD-1/PD-L1 therapy [40].

Maximizing this dual benefit requires refined consideration of therapeutic timing. A key clinical question is whether intervention should be prophylactic (e.g., at diagnosis) or therapeutic (upon onset of cachexia or immunotherapy resistance). Preclinical models suggest that early GDF15 blockade may be more effective at preventing the establishment of irreversible muscle wasting and T cell exhaustion programs [62]. Furthermore, given the feedback increase in GDF15 following chemotherapy, sequencing GDF15 axis inhibition with chemotherapy cycles may potentially protect host physiology while counteracting chemotherapy-induced immunosuppression [88]. Thus, future clinical trial design must evolve beyond simple combination therapy toward chronological and adaptive strategies that are informed by dynamic biomarker profiles.

### 6.3. Future Frontiers: Neuromodulation and Biomarker-Driven Personalization

To fully realize the potential of the GDF15-GFRAL axis, future research must address several frontiers.

First, a deeper understanding of the neural circuits downstream of GFRAL is needed [25]. While antibodies systemically block signaling, targeted neuromodulation through chemogenetics or focused pharmacotherapy could potentially dissociate the beneficial metabolic effects from aversive nausea, optimizing tolerability [54].

Second, biomarker-driven patient stratification will be crucial for personalization. GDF15 expression and signaling are highly context-dependent and are influenced by tumor genetics (e.g., tumor protein p53 (TP53), phosphatidylinositol-4,5-bisphosphate 3-kinase catalytic subunit alpha (PIK3CA), and STK11 status), tumor type, and host factors [104,114]. Circulating GDF15 levels may serve as a predictive biomarker for cachexia risk or treatment response. However, biomarker utility must be scrutinized across study populations, as evidenced by racial differences in the predictive value of GDF15 for cachexia in pancreatic cancer [117]. Future trials should stratify patients based on molecular profiles and GDF15 pathway activity to identify optimal responders.

Third, the clinical application of GDF15 is complicated by its functional diversity and context dependence (Table 2). The intracellular uncleaved form of GDF15 (pro-NAG-1) can bind to epithelial cell adhesion molecule (EpCAM) and suppress the β-catenin and NF-κB pathways, acting as a tumor suppressor in early-stage CRC [118]. Furthermore, GDF15 exhibits context-dependent, protective roles in non-cancer disease states; for instance, it may improve hepatic steatosis by mitigating oxidative stress and inflammasome activation. This contrasts with its more widely recognized role in disease progression, illustrating how GDF15 function varies with molecular form, cellular location, cancer type, and disease stage. For these reasons, GDF15 should not be used alone as a universal biomarker. Future efforts should combine GDF15 measurements with those of other biomarkers, such as specific GDF15 isoforms, tumor genetic features, and patient comorbidities, to improve diagnostic and prognostic accuracy.

### 6.4. Positioning GDF15 Targeting Within the Immunotherapeutic Landscape

The advent of GDF15-GFRAL axis inhibitors necessitates a clear understanding of their position within the existing arsenal of cancer immunotherapies.

#### 6.4.1. A Distinct Niche

Unlike classical pro-inflammatory cytokines, such as IL-6 or TNF-α, which primarily act as direct immune activators or inflammatory amplifiers, GDF15 functions as a metabolic immune checkpoint. While IL-6 blockade can ameliorate cachexia in specific contexts, its effects in solid tumors are less defined and may inadvertently suppress beneficial anti-tumor immunity. Similarly, TGF-β inhibitors face challenges with on-target toxicity due to TGF-β’s pleiotropic roles in homeostasis. GDF15 targeting offers a more selective approach, specifically disrupting a pathway that causes central anorexia and at the same time enforces local T cell exclusion/dysfunction via LFA-1 blockade and Treg expansion.

#### 6.4.2. A Predictive Biomarker for Immunotherapy

High pre-treatment GDF15 levels may identify patients likely to exhibit primary resistance to PD-1/PD-L1 checkpoint inhibitors, since GDF15 directly impedes T cell recruitment, which is a prerequisite for ICI efficacy. Therefore, GDF15 may serve as a negative predictive biomarker for single-agent ICI therapy and positively predict the response to GDF15-axis inhibitors. This positions GDF15 for biomarker-driven stratification, where patients with high GDF15 levels could be directed toward trials involving GDF15 blockade plus ICI treatment, while those with low GDF15 may proceed with standard ICI therapy.

Beyond the tumor, this strategy holds promise for mitigating the broader metabolic consequences of cancer, such as insulin resistance and bone loss, which are often exacerbated by both the disease and its treatments. Thus, GDF15 targeting represents an integrated approach to managing cancer as a systemic metabolic disease.

#### 6.4.3. Rational Combinations

GDF15 targeting is not envisioned as a standalone replacement for existing immunotherapies but as a powerful complementary strategy. GDF15 neutralization with immune checkpoint blockade removes the “brakes” on T cell infiltration, increasing the pool of tumor-infiltrating lymphocytes that can be released by anti-PD-1/PD-L1 therapy, potentially reversing ICI resistance in “immunologically cold” tumors. Currently, the combination of GDF15 neutralization with ICIs is considered the most clinically realistic and immediately translatable approach for advanced malignancies, as it capitalizes on existing immunotherapeutic infrastructure while directly removing physical barriers to T-cell infiltration. Combining GDF15 inhibitors in the context of cachexia may serve as a metabolic primer, improving the host’s physical condition to better withstand and respond to subsequent therapies. With metabolic or chemotherapeutic agents, combining GDF15 inhibitors with drugs that induce tumor cell stress could preempt the surge in GDF15 that contributes to resistance and cachexia, protecting the host while improving anti-tumor efficacy.

In conclusion, GDF15-GFRAL axis inhibition occupies a unique niche by concurrently targeting a systemic metabolic disorder (cachexia) and a specific local immunosuppressive mechanism. Its value as a predictive biomarker and its rational combination with ICIs establishes it as a next-generation strategy to extend the benefits of immunotherapy to a broader patient population.

### 6.5. Clinical Translation: A Practical Guide for GDF15-Targeted Therapy

Practical guidance for patient selection and treatment monitoring becomes increasingly relevant as GDF15-targeting agents advance through clinical development.

**i. When to suspect GDF15-driven cachexia.** This condition should be suspected in patients with advanced solid tumors (e.g., pancreatic, lung, colorectal, and HCC) who present with unexplained progressive weight loss (>5% over 6 months) despite adequate caloric intake, reduced appetite or early satiety, fatigue and sarcopenia, and elevated circulating GDF15 levels (>1200 pg/mL in some cohorts) [119] in the absence of other obvious causes. GDF15 should be suspected especially when cachexia coincides with poor response to ICIs, as high GDF15 levels predict primary resistance to anti-PD-1/PD-L1 therapy.

**ii. Interpreting circulating GDF15 levels.** Although no universally accepted cutoff exists, GDF15 levels > 1500–2000 pg/mL are commonly associated with cancer cachexia and poor prognosis and should be interpreted in the context of tumor type, stage, and inflammatory status. Serial measurements may help to assess the response to therapy. A decline in GDF15 level after tumor-directed treatment may indicate reduced tumor burden, whereas persistence or increase suggests treatment resistance or progressive cachexia [30]. GDF15 should not be used alone. Combining with inflammatory markers (IL-6, CRP), nutritional markers (albumin, prealbumin), and tumor genetic data (e.g., TP53, STK11, and KRAS status) improves specificity for patient selection.

**iii. Which patients might benefit from GDF15-targeted therapy?** Ideal candidates include patients with radiologically measurable disease and evidence of cachexia. This includes individuals with elevated circulating GDF15 levels (>1500 pg/mL) and/or tumor GDF15 overexpression demonstrated by immunohistochemistry analysis, patients with molecular subtypes known to upregulate GDF15 (TP53 mutant, STK11/LKB1-mutant non-small cell lung cancer, KEAP1/Nrf2-activated tumors, and tumors with high oxidative or ER stress), and those experiencing primary resistance to ICIs.

**iv. Practical considerations for combining GDF15 blockade with ICIs.** Preclinical evidence suggests that early intervention at diagnosis or before cachexia onset may prevent irreversible wasting and T cell exhaustion. In the clinic, a GDF15-neutralizing antibody may be added when initiating ICI therapy in high-risk patients [44]. Because GDF15 level rises after chemotherapy, sequencing GDF15 blockade between chemotherapy cycles could protect host metabolism while enhancing immune recovery. Monitoring should include body weight, appetite (visual analogue scale), lean body mass (computed tomography-based sarcopenia assessment), and circulating GDF15 levels at baseline and every 2–4 weeks, with immune-related adverse events monitored according to standard ICI guidelines. GDF15 blockade pairs logically with anti-PD-1/PD-L1 agents. Future trials may also explore combinations with chemotherapy, anti-cytotoxic T-lymphocyte antigen-4, or metabolic modulators (e.g., β-blockers and GLP-1 analogs) based on molecular rationale.

### 6.6. Limitations and Translational Barriers

Despite the therapeutic promise, translating GDF15-GFRAL axis inhibitors into clinical practice faces several critical limitations and translational barriers. A primary challenge is the lack of universally established threshold levels for circulating GDF15 to definitively stratify patients for therapy. Furthermore, the complexities of context-dependent receptor signaling in the TME complicate universal targeting strategies. Physiologically, GDF15 is an important mediator of tissue tolerance and protective emetic reflexes against toxins, including chemotherapeutics. Consequently, there is a theoretical risk that long-term, potent GDF15 neutralization could inadvertently impair the host’s natural aversive responses to noxious stimuli or reduce systemic tolerance to inflammation. Finally, optimizing the therapeutic sequencing of GDF15 blockade with standard cyclic chemotherapies to balance immune restoration with physiological protection remains a critical barrier requiring further prospective clinical evaluation.

## 7. Conclusions and Prospects

The present review explores converging evidence to establish GDF15 as a pivotal meta-hormone co-opted in cancer. Under persistent tumor-derived stress, GDF15 is transformed from a physiological stress sensor into a pathological orchestrator of a dual offensive. Systemically, it exploits the brain–body axis via GFRAL to drive cachexia, leading to the devastating loss of skeletal muscle and adipose tissue. Locally, it becomes the architect of immunosuppression within the TME. These pathways are dynamically interlinked, forming a self-reinforcing cycle that explains the lethal synergy between wasting, immune dysfunction, and therapeutic failure.

Therapeutically, recalibrating the GDF15-GFRAL axis emerges as a rational strategy with a unique dual-benefit potential. It may be possible to simultaneously ameliorate cachexia by disrupting this central hub, thereby preserving muscle mass, physical function, and patient quality of life, and dismantle immunosuppression to potentiate anti-tumor immunity. This approach is distinct from, yet highly complementary to, established immunotherapies. The potential of GDF15 as a biomarker for patient stratification further enhances its translational relevance. While the use of neutralizing antibodies and receptor blockers is advancing clinically, future success will depend on biomarker-driven classification, downstream neural circuitry exploration, and rational combination therapies.

## Figures and Tables

**Table 1:** Molecular Subtypes and GDF15-Driven Pathologies: Toward Precision Targeting.

Molecular Subtype	Key Driver(s)/Pathway	GDF15 Regulation Mechanism	Immunosuppressive Signature	Cachexia Risk	Precision Therapeutic Strategy	Clinical Evidence/Refs
p53/Genotoxic Stress	TP53 mutation, DNA damage response	Transcriptional activation via p53 response elements	T cell dysfunction, reduced CD8^+^ infiltration	Moderate	GDF15 neutralizing antibody + chemotherapy (e.g., cisplatin, oxaliplatin)	Tuval et al., 2024 [6]; Abdul Razak et al., 2020 [9]
STK11/LKB1 Deficient	“STK11/LKB1” loss, AMPK/mTOR dysregulation	Metabolic stress induced GDF15 via ISR/ATF4	Immunologically “cold” TME, low PD-L1, excluded CD8^+^ T cells	High	GDF15 antibody + anti PD-1/PD-L1 (overcome ICI resistance)	Sjøberg et al., 2023 [100]; Yu et al., 2026 [25]
Nrf2 Activated/Oxidative Stress	KEAP1 mutation, Nrf2 stabilization	GDF15-Nrf2 reciprocal feedback loop; redox homeostasis	M2-macrophage polarization, Treg expansion	High	GDF15 antibody + Nrf2 inhibitor? (preclinical); combine with antioxidants	Lin et al., 2024 [88]; Kang et al., 2021 [10]
Inflammatory/NF-κB Driven	Chronic inflammation, NF-κB activation	Inflammatory cytokine induced GDF15 (IL-6, TNF-α)	Treg expansion, myeloid derived suppressor cells	High	GDF15 antibody + anti IL-6 (e.g., tocilizumab) or JAK inhibitors	Luan et al., 2019 [13]; Reyes & Yap, 2023 [101]
MAPK/PI3K Activated	EGFR, KRAS, PIKCA mutations	EGR1-GDF15 positive feedback; downstream AKT/ERK signaling	T cell exclusion via LFA-1/ICAM-1 blockade	Moderate	GDF15 antibody + MAPK/PI3K pathway inhibitors (e.g., trametinib)	Jin et al., 2021 [75]; Melero et al., 2025 [40]
GDF15 High (by IHC or serum)	Tumor intrinsic or stromal production	High baseline GDF15 expression (epigenetic, TME stressors)	Treg^+^, M2^+^, exhausted CD8^+^ T cells	High	GDF15 antibody monotherapy (if cachexia dominant) or + ICI (if immune resistance)	Wischhusen et al., 2020 [30]; Haake et al., 2023 [41]
Therapy Induced Persister State	Chemotherapy (cisplatin, gemcitabine), targeted agents	Stress induced GDF15 in drug tolerant persister cells	Immunosuppressive TME, resistance to ICIs	Variable	Time sequenced GDF15 blockade between chemotherapy cycles	Izaguirre et al., 2020 [76]; Bellio et al., 2022 [77]

**Table 2:** Targeting the GDF15 GFRAL Axis: Clinical Landscape and Molecular Insights.

Agent	Mechanism of Action	Tumor Type Evaluated	Primary Endpoint Measured (Cachexia vs. Immune)	Development Stage	Key Findings/Efficacy Signals	Biomarker Strategy	Combination Partners	Limitations/Challenges	Refs/NCT ID
Ponsegromab (PF 06946860)	Humanized mAb against GDF15; neutralizes circulating ligand	NSCLC, pancreatic cancer, CRC	Cachexia (body weight, appetite, physical activity)	Phase II	Increased body weight, appetite, and physical activity; reduced cachexia symptoms in GDF15 high patients	Baseline serum GDF15 (>1500 pg/mL); on treatment GDF15 reduction	None (monotherapy in cachexia); potential + ICI in future	Long term efficacy on survival; optimal duration	NCT05546476; Groarke et al., 2024 [110]; Fillon, 2025 [109]
Visugromab (CTL 002)	Humanized mAb against GDF15; ligand neutralization	Advanced solid tumors (NSCLC, HCC, bladder)	Immune Response (ICI resistance reversal, CD8^+^ T cell infiltration)	Phase II	Reverses ICI resistance in “cold” tumors; enhances CD8^+^ T cell infiltration	Tumor GDF15 IHC; serum GDF15; PD-L1 expression	Anti PD-1 (nivolumab) + chemotherapy	Durable responses in subset only; need predictive biomarkers	NCT04725474; NCT07246863; Melero et al., 2025 [40]
NGM120	mAb against GFRAL; blocks GDF15-GFRAL interaction	Pancreatic cancer, advanced solid tumors	Cachexia/General Efficacy (Tolerability)	Phase I	Well tolerated; preliminary signal in combination with gemcitabine + nab paclitaxel	GFRAL expression (brain only limits tissue biopsy utility)	Gemcitabine/nab paclitaxel; anti PD-1	Peripheral GFRAL? brain penetration not required for efficacy	NCT04068896
AZD8853	Humanized mAb against GDF15; high affinity ligand neutralization	NSCLC, MSS CRC, urothelial cancer	Immune/Anti-tumor Response	Phase I	Favorable safety; only transient GDF15 suppression; limited durable antitumor activity as monotherapy	Serum GDF15; CD8^+^ PET imaging (substudy)	None (monotherapy)	Transient target suppression; requires optimized dosing or combination	NCT05397171; Carneiro et al., 2025 [111]
AV 380	Humanized mAb against GDF15	Cancer cachexia models (unspecified solid tumors)	Cachexia	Phase I	Ongoing; preclinical efficacy in cachexia models	Serum GDF15	None (monotherapy)	Clinical data pending	NCT05865535
GFS202A	mAb against GDF15	Cancer cachexia (unspecified)	Cachexia	Phase I	Ongoing	Serum GDF15	None (monotherapy)	Clinical data pending	NCT06898255
GB18 06	Nanobody against GDF15; high potency	Preclinical cancer models	Cachexia (weight loss, physical function)	Preclinical	Reversed weight loss; improved physical function	Murine GDF15	None	Requires clinical translation	Huang et al., 2024 [63]
GFRAL Fc	Decoy receptor; traps GDF15	HCC (Preclinical models)	Immune Response (TME remodeling, PD-1 efficacy)	Preclinical	Enhanced anti PD-1 efficacy; remodeled TME	Murine GDF15	Anti PD-1	Clinical development needed	Shi et al., 2025 [73]
Small molecule GFRAL antagonists	Oral bioavailable; brain penetrant GFRAL blockade	Preclinical models	Cachexia (anorexia vs. nausea)	Discovery/Preclinical	Potential to dissociate anorexia from nausea	–	–	Need structure based drug design	Borner et al., 2023 [107]

## Data Availability

No new data were created or analyzed in this study. Data sharing is not applicable to this article.

## Abbreviations

The following abbreviations are used in this manuscript:

AKT	protein kinase B
AMPK	AMP-activated protein kinase
AP-1	activator protein 1
ATGL	adipose triglyceride lipase
BET	bromodomain and extra-terminal domain family
CAFs	cancer-associated fibroblasts
CD48	cluster of differentiation 48
CHIP	STIP1 homology and U-box containing protein 1
CRC	colorectal cancer
CREB	cAMP response element-binding protein
CTLA-4	cytotoxic T-lymphocyte antigen-4
DC	dendritic cell
EGR1	early growth response 1
EpCAM	epithelial cell adhesion molecule
ER	endoplasmic reticulum
ERK	extracellular signal-regulated kinase
FOXP3	Forkhead box P3
GDF15	growth differentiation factor 15
GFRAL	glial cell line-derived neurotrophic factor family receptor alpha-like
GLUT-1	glucose transporter 1
GSK3β	glycogen synthase kinase 3 beta
HCC	hepatocellular carcinoma
HIF-1α	hypoxia-inducible factor 1 alpha
HK2	hexokinase 2
HSL	hormone-sensitive lipase
ICAM-1	intercellular adhesion molecule 1
ICIs	immune checkpoint inhibitors
IHC	immunohistochemistry
IL-6	interleukin 6
IRE1α	inositol-requiring enzyme 1 alpha
LDHA	lactate dehydrogenase
LFA-1	lymphocyte function-associated antigen 1
LKB1	liver kinase B1
mAb	monoclonal antibody
MAPK	mitogen-activated protein kinase
MEK	mitogen-activated protein kinase kinase
mTOR	mechanistic target of rapamycin
NF-κB	nuclear factor kappa-light-chain-enhancer of activated B cell
Nrf2	nuclear factor erythroid 2-related factor 2
NSCLC	non-small cell lung cancer
PD-1	programmed cell death protein 1
PD-L1	programmed death-ligand 1
PFK	phosphofructokinase
PI3K	phosphoinositide 3-kinase
PIK3CA	phosphatidylinositol-4,5-bisphosphate 3-kinase catalytic subunit alpha
PKA	protein kinase A
RET	rearranged during transfection
ROS	reactive oxygen species
SMAD2/3	mothers against decapentaplegic homolog 2 and 3
STK11	serine/threonine kinase 11
TAMs	tumor-associated macrophages
TGF-β	transforming growth factor beta
TGFBR2	transforming growth factor beta receptor 2
TME	tumor microenvironment
TNF-α	tumor necrosis factor alpha
TP53	tumor protein p53
Treg	regulatory T cell
XBP1	X-box binding protein 1
